# Effects of 1α,25-dihydroxyvitamin D_3_ and tacalcitol on cell signaling and anchorage-independent growth in T98G and U251 glioblastoma cells

**DOI:** 10.1016/j.bbrep.2022.101313

**Published:** 2022-07-31

**Authors:** Frida Olsson, Niki Sarri, Natalia Papadopoulos, Johan Lennartsson, Maria Norlin

**Affiliations:** a Department of Pharmaceutical Biosciences, Uppsala University, Uppsala, Sweden; b Department of Medical Biochemistry and Microbiology, Uppsala University, Uppsala, Sweden

**Keywords:** 1,25-Dihydroxyvitamin D, Tacalcitol, Vitamin D analog, Glioblastoma, Cell signaling, Tumorigenicity, ERK, extracellular signal-regulated kinase, PI3K, phosphatidylinositol 3-kinase, PLC, phospholipase C, STAT3, signal transducer and activator of transcription-3, VDR, vitamin D receptor

## Abstract

The active hormonal form of vitamin D, 1α,25-dihydroxyvitamin D_3_, is reported to have 1000s of biological targets. The growth-suppressive properties of 1α,25-dihydroxyvitamin D_3_ and its synthetic analogs have attracted interest for the development of treatment and/or prevention of cancer. We examined effects of 1α,25-dihydroxyvitamin D_3_ and the vitamin D analog tacalcitol on signaling pathways and anchorage-independent growth in T98G and U251 glioblastoma cells. Assay of signaling proteins important for cellular growth indicated suppression of p70-S6 kinase levels by 1α,25-dihydroxyvitamin D_3_ and tacalcitol in T98G cells, whereas the levels of PLCγ, a target for phospholipid signaling, was slightly increased.

Activation of STAT3, an important regulator of malignancy, was suppressed by 1α,25-dihydroxyvitamin D_3_ and tacalcitol in T98G and U251 cells. However, despite the close structural similarity of these compounds, suppression was stronger by tacalcitol (1α,24-dihydroxyvitamin D_3_), indicating that even minor modifications of a vitamin D analog can impact its effects on signaling. Experiments using soft agar colony formation assay in T98G and U251 cells revealed significant suppression by 1α,25-dihydroxyvitamin D_3_ and tacalcitol on anchorage-independent growth, a property for cancer invasion and metastasis known to correlate with tumorigenicity. These findings indicate that vitamin D and its analogs may be able to counteract the oncogenic transformation, invasion and metastatic potential of glioblastoma and prompt further study of these compounds in the development of improved therapy for brain cancer.

## Highlights

• Vitamin D and its analog tacalcitol affect signaling in glioblastoma cell lines.
• Suppression of the oncogenic STAT-3 is stronger by tacalcitol than by vitamin D.
• Tacalcitol and vitamin D inhibit anchorage-independent growth in T98G and U251 cells.
• Vitamin D analogs may be useful to combat oncogenicity and invasion of glioblastoma.

## 1 Introduction

Vitamin D is important for human physiological function in several ways. In addition to its well-known roles in the maintenance of calcium levels and bone health, the vitamin D hormone affects a number of additional systems and processes, including e. g. neurotransmission, immune function and cellular growth and survival [1]. The inactive form of vitamin D (cholecalciferol), obtained in the diet or synthesized in the skin when exposed to UV radiation, requires enzymatic activation by two subsequent hydroxylation steps to produce its active hormonal form, 1α,25-dihydroxyvitamin D_3_ (also called calcitriol) [1]. 1α,25-dihydroxyvitamin D_3_ can bind to the vitamin D receptor (VDR) which functions as a transcription factor and is reported to affect 1000s of target genes, linked to a variety of biological functions [[2], [3], [4]]. 1α,25-Dihydroxyvitamin D_3_ has also been reported to act independently of VDR, via other receptors and/or pathways [[4], [5], [6]]. Alternative pathways of vitamin D activation have been described, producing other biologically active hydroxyderivatives [7]. 1α,25-Dihydroxyvitamin D_3_ increases differentiation and suppresses proliferation in many cell types and under some conditions this compound may also affect cellular viability [1,8]. The growth-suppressive properties of 1α,25-dihydroxyvitamin D_3_ have attracted considerable interest for its potential in development of treatment and/or prevention of cancer [2,3,5,8]. Many studies *in vitro* and *in vivo* have reported suppression by 1α,25-dihydroxyvitamin D_3_ on malignant growth of e.g. prostate, ovary and colon [2,9]. Furthermore, vitamin D status is associated with the risk of developing cancer [8,9]. Unfortunately, the concentration of 1α,25-dihydroxyvitamin D_3_ required for substantial growth suppression is generally too high for systemic administration without risking adverse side effects such as hypercalcemia [10,11]. Therefore, in order to exploit the properties of vitamin D for the development of improved cancer therapy, a large number of vitamin D analogs with different chemical structures have been synthesized, in attempts to obtain more efficient growth suppression and less risk of side effects [[10], [11], [12]]. Vitamin D analogs are used clinically, e. g. in the treatment of psoriasis, and some preclinical studies in animals suggest promising effects by some of these compounds in experimentally induced tumors [13,14]. However, the influence of the synthetic vitamin D analogs may vary between different cell types and their effects on signaling pathways in the cells remain to a large extent unclear.

**Fig. 1:**
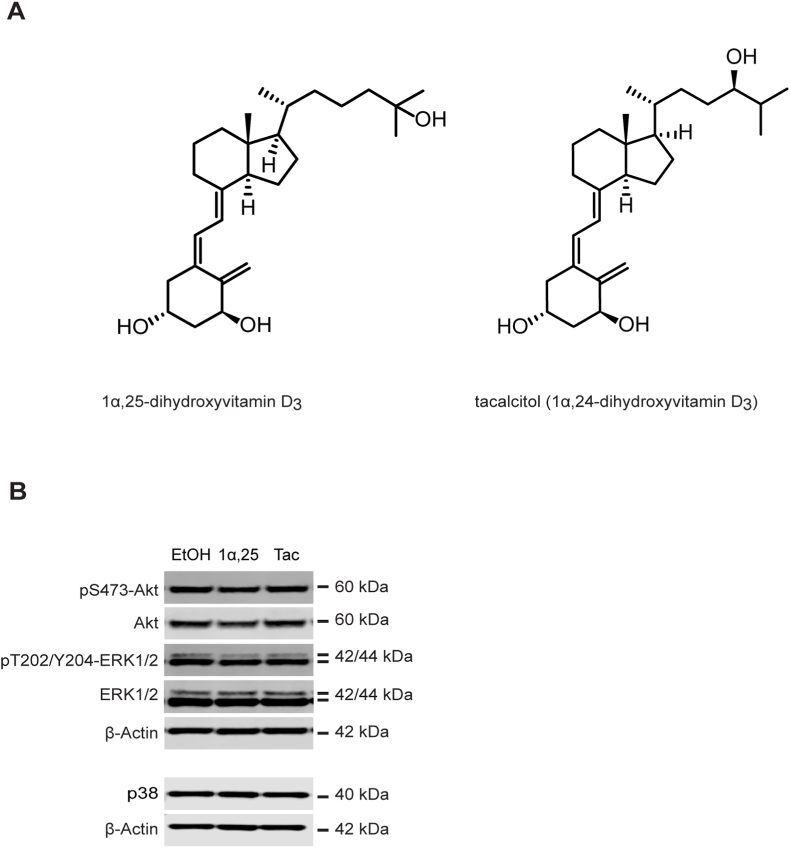
(**A**) The structures of 1α,25-dihydroxyvitamin D_3_ and the vitamin D analog tacalcitol (1α,24R-dihydroxyvitamin D_3_). (**B**) Western blot analysis of pS473-Akt, Akt, pT202/Y204-ERK1/2, ERK1/2 and p38 in lysates from T98G cells after treatment with 1 μM of 1α,25-dihydroxyvitamin D_3_, (1α,25), 1 μM of tacalcitol (Tac) or vehicle (EtOH) for 24 h. β-Actin was used as the loading control. The experiment was repeated independently four times. A representative experiment is shown.

Previously, we reported the suppressive effects of 1α,25-dihydroxyvitamin D_3_ and the vitamin D analog tacalcitol (1α,24-dihydroxyvitamin D_3_) (Fig. 1A) on the growth and migration of human T98G cells, a cell model often used to study glioblastoma [15]. Glioblastoma is the most common primary brain tumor. This form of cancer grows rapidly, is invasive and is known for its poor prognosis, its mortality rate (less than 10% survival at 5 years from onset) and its lack of efficient treatment [16].

In the present study we explored the effects of tacalcitol and 1α,25-dihydroxyvitamin D_3_ on signaling pathways and anchorage-independent growth in human glioblastoma cells T98G and U251. The findings of the present study indicate that the cellular response to these two molecules, despite their close resemblance in structure, is partly different. In addition, we report significant suppression of anchorage-independent growth of T98G and U251 cells by tacalcitol and 1α,25-dihydroxyvitamin D_3_, supporting a functional role for these compounds in decreasing tumorigenicity of glioblastoma.

## 2 Materials and methods

### 2.1 Chemicals

1α,25-Dihydroxyvitamin D_3_ (sc-202877A, Santa Cruz Biotechnology) and tacalcitol (4157, TOCRIS Bioscience) were stored as stock solutions in – 20 °C, dissolved in toluene and pure ethanol 1:1. The toluene was removed prior to experimentation and the compounds were added to the cell cultures in ethanol. In all experiments, the final ethanol concentration was kept below 0.1% (v/v).

### 2.2 Cell culture

Human T98G and U251 glioblastoma cells were cultured in DMEM + GlutaMAX (31966, Thermo Scientific) and RPMI 1640 + Glutamax (61870, Thermo Scientific) respectively, supplemented with 10% (v/v) fetal bovine serum (FBS, 10500–064, Thermo Scientific).

### 2.3 Western blotting

For Western blot experiments, the cells were seeded in 6-well plates at a density of 25 000 cells/cm^2^ one day prior to treatment. Cells were treated with ethanol, 1 μM 1α,25-dihydroxyvitamin D_3_ or 1 μM tacalcitol for 24 h. Cells were then rinsed with ice-cold Dulbecco's phosphate-buffered saline (14190–169, Thermo Scientific) and lysed with a buffer containing 20 mM Tris HCl pH 8, 137 mM NaCl, 10% glycerol, 1% Nonidet P-40, 2 mM EDTA and 0.1% SDS, supplemented with a protease and phosphatase inhibitor cocktail (PhosSTOP, 4906837001 and cOmplete, 4693159001, Sigma). After centrifugation at 12 000 g for 15 min, the supernatant was collected as whole cell lysate and 1 × NuPAGE™ LDS Sample Buffer, 50 mM dithiothreitol (DTT) was added. Samples were denatured at 95 °C for 5 min before loading on SDS-polyacrylamide gels for electrophoresis. Chameleon Duo Pre-Stained Protein Ladder (LI-COR Biosciences) was used for size reference. After electrophoresis, protein was transferred to PVDF-FL membranes. Membranes were blocked for 2 h at room temperature in Intercept blocking buffer (LI-COR Biosciences, diluted 1:3 in TBS) and incubated at 4 °C over-night with primary antibodies. The following primary antibodies were used: rabbit anti-pY783-PLCγ1 (#2821, 1:1000, Cell Signaling Technology), mouse anti-PLCγ1 (H00005335-M01, 1:1000, Novus Biologicals), mouse anti-pThr389-p70-S6 Kinase (#7053, 1:1000, Cell Signaling Technology), rabbit anti-p70-S6 Kinase (#7053, 1:1000, Cell Signaling Technology), rabbit anti-pY705-STAT3 (#9145, 1:500, Cell Signaling Technology), mouse anti-STAT3 (#9139, 1:1000, Cell Signaling Technology), rabbit anti-pS473-AKT (#4060, 1:500, Cell Signaling Technologies), mouse anti-AKT (#2920, 1:500 Cell Signaling Technologies), rabbit anti-pT202/Y204-ERK1/2 (#9101, 1:1000, Cell Signaling Technology), rabbit anti-ERK1/2 (#4695, 1:1000, Cell Signaling Technology), mouse anti-β-Actin (sc-47778, 1:1000, Santa Cruz Biotechnology), mouse anti-pT180/182-p38 (#9216, 1:1000, Cell Signaling Technology), rabbit anti-p38 (#9212, 1:1000, Cell Signaling Technology) and rabbit anti-β-Actin (ab8227, 1:1000, Abcam). The membranes were washed 3 × 10 min in 0.05% Tween-20 in TBS both before and after incubation for 1 h with secondary antibodies (Donkey anti-rabbit IRDye800, #926–32213,1:10 000, Licor or Donkey anti-mouse, #A10038, 1:10 000, Thermo Scientific). The membranes were scanned and protein expression levels were analyzed using an Odyssey Scanner and ImageStudio v5.2.5 software (both from LI-COR Biosciences). β-Actin was used as loading control.

### 2.4 RNA extraction and real-time qPCR

T98G cells were incubated with ethanol or 1 μM tacalcitol for 24 h followed by RNA isolation using RNeasy mini kit (Qiagen). cDNAs were synthesized with qPCRBIO cDNA Synthesis Kit (PCR Biosystems) and used for real-time PCR amplification with qPCRBIO SyGreen Mix (PCR Biosystems) on a CFX Connect Real-Time PCR System (Bio-Rad). β-Actin, TBP (TATA-binding protein) and 18S were used as control (housekeeping) genes. Forward and reverse primers were as follows: 5′-TCTGCCGGAGAAACAGTTGG-3′ (forward) and 5′-AGGTACCGTGTGTCAAGCTG-3′ (reverse) for STAT3; 5′-TCTACAATGAGCTGCGTGTG-3′ (forward) and 5′-AGCCTGGATAGCAACGTACA-3′ (reverse) for β-actin; 5′-TGCACAGGAGCCGCCAAGAGTGA-3' (forward) and 5′-CACATCACAGCTCCCCACCA-3' (reverse) for TBP; 5′-AGTCCTGCCCTTTGTACACA-3' (forward) and 5′GATCCGAGGGCCTCACTAAAC3' (reverse) for 18S.

Each PCR was done in triplicate and the experiment was repeated four times. Data were analyzed using the 2^−ΔΔCT^ method.

### 2.5 STAT3 Transcription Factor Assay

Activation of STAT3 in T98G and U251 cells was measured by a colorimetric ELISA-based STAT3 assay kit (STAT3 Transcription Factor Assay kit, ab207229, Abcam). Cells were cultured in T175 flasks (25 000 cells/cm^2^) and treated for 24 h with ethanol, 1α,25-dihydroxyvitamin D_3_ or tacalcitol at the indicated concentrations. Preparation of nuclear cell lysates and colorimetric STAT3 detection was carried out according to the manufacturer's protocol. A BCA kit (sc-202389, ChemCruz) was used to measure the protein concentration. The absorbance, proportional to activation of STAT3, was measured at OD 450 nm using a FLUO STAR Omega microplate reader (BMG LABTECH).

### 2.6 Soft agar colony formation assay

Cells were trypsinized and re-suspended (2000 cells/cm^2^) in medium containing 0.3% agarose (16520050, Invitrogen) and 3% (for T98G) or 10% (for U251) FBS. Cell suspensions were treated with ethanol, 1α,25-dihydroxyvitamin D_3_ (0.2–5 μM) or tacalcitol (0.2–5 μM) and placed on a 0.6% agarose layer in a 48 well plate. Each treatment condition was tested in triplicate. Cells were cultured for three weeks with addition of new ethanol, 1α,25-dihydroxyvitamin D_3_ or tacalcitol in fresh media every 72 h, followed by counting the number of formed T98G (≥70 μm) or U251 (≥40 μm) colonies.

### 2.7 Statistical analysis

Experiments were repeated independently at least three times. Shapiro-Wilk test was performed for all experiments to test for normal distribution. Significance was determined by one way ANOVA for soft agar colony formation assay and by Student's t-test (two-tailed, unpaired) for Western blotting.

For quantification using STAT3 Transcription Factor Assay, data are expressed as average values relative to the control ± SD. The values of experimental samples were divided by the value of control sample for each experiment and one sample Student's t-test was performed to test whether the average differs significantly from the control.

P-values *<0.05, **<0.01, ***<0.001 and ****<0.0001 were considered significant.

## 3 Results and discussion

### 3.1 Effects by tacalcitol and 1α,25-dihydroxyvitamin D_3_ on signaling proteins in T98G cells

**Fig. 2:**
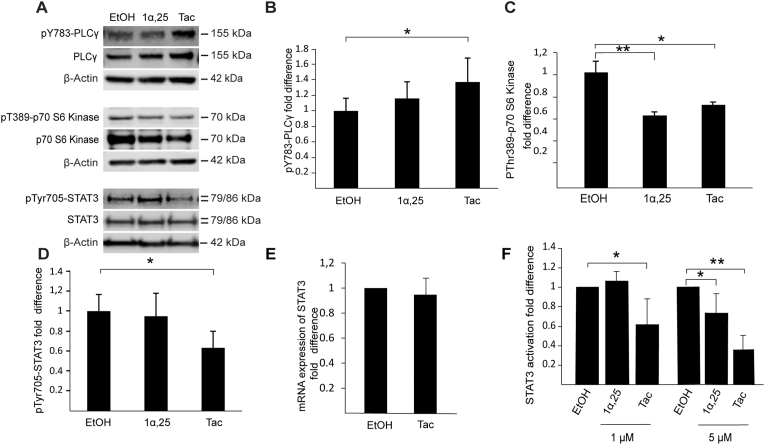
(**A**) Western blot analysis of pY783-PLCγ, PLCγ, pT389-p70-S6 kinase, p70-S6 kinase, pY705-STAT3 and STAT3 after treating T98G cells with 1 μM of 1α,25-dihydroxyvitamin D_3_ (1α,25), 1 μM of tacalcitol (Tac) or vehicle (EtOH) for 24 h. (**B**), (**C**) and (**D**) Quantitative analysis of the levels of pY783-PLCγ, pT389-p70-S6 kinase and pY705-STAT3 inT98G cell lysates in the treatment groups as compared to vehicle. β-Actin was used as loading control for pY783-PLCγ and pT389-p70-S6. pY705-STAT3 was normalized to total STAT3. The values are presented as mean ± standard deviation of at least four independent determinations and shown as fold difference compared to ethanol-treated cells. (**E**) Analysis of STAT3 mRNA in T98G cells treated with tacalcitol (1 μM, 24h) compared to vehicle-treated cells. The graph shows the means ± standard deviation of four independent experiments. (**F**) Determination of STAT3 activation in T98G cells using a STAT3 assay kit. STAT3 activation was measured in cells cultured for 24 h in the absence (EtOH) and presence of 1α,25-dihydroxyvitamin D_3_ (1α,25) or tacalcitol (Tac) in the indicated concentrations. The values are presented as mean ± standard deviation of five independent determinations. * = p < 0.05.

In order to explore the cell signaling mechanisms affected by tacalcitol and 1α,25-dihydroxyvitamin D_3_ (structural comparison Fig. 1A) in T98G glioblastoma cells, we assayed potential effects of these compounds on different signaling pathways, using Western blotting. Considering the potential for vitamin D and its analogs in the treatment of cancer, we first focused our interest on pathways involved in cellular growth and survival such as the ERK, p38 and Akt/PI3K pathways. We did not observe any significant effect by these compounds on the levels of Akt, phospho-Akt, ERK, phospho-ERK or p38 in T98G cells (Fig. 1B). The phosphorylated form of p38 was also examined but not detectable in T98G cells under these conditions (data not shown). We observed, however, suppressed levels of phosphorylated p70-S6 kinase (Fig. 2A and C), a protein target of several signaling pathways, including PI3K/Akt, known to induce protein synthesis and cellular proliferation. In contrast, a small but significant increase was observed for phosphorylated PLCγ, which is a target protein for signal transduction involving membrane phospholipids (Fig. 2A and B). PLCγ is involved in several processes, including e. g. cellular growth and brain development [17]. Studies have indicated effects of PLCγ in tumorigenesis but the potential role of this protein in cancer progression is unclear [18]. The effect on PLCγ was somewhat stronger for tacalcitol than for 1α,25-dihydroxyvitamin D_3_. For both p70-S6 kinase and PLCγ we observed effects also on the unphosphorylated forms of both these proteins, suggesting that their basal levels rather than their activation might be influenced by tacalcitol and 1α,25-dihydroxyvitamin D_3_. Furthermore, at a concentration of 1 μM we observed marked suppression of phosphorylated STAT3 by tacalcitol but not by 1α,25-dihydroxyvitamin D_3_, indicating a difference in cellular response to these structurally very similar compounds (Fig. 2D). As observed in the Western blotting experiments, unphosphorylated STAT3 was not altered by either of the treatments, indicating a specific effect by tacalcitol on STAT3 activation under these conditions. We also carried out real-time PCR experiments in cells treated with tacalcitol to examine the basal level of STAT-3 expression (Fig. 2E). These experiments showed no effect by tacalcitol on STAT3 mRNA levels, confirming that the observed effect is specifically on the activation of STAT3.

### 3.2 Differential effects of 1α,25-dihydroxyvitamin D_3_ and tacalcitol on STAT3 activation in T98G cells

In order to further study the effects of tacalcitol and 1α,25-dihydroxyvitamin D_3_ on STAT3, a protein well-known for its oncogenic potential, we analyzed the effects of these two compounds in T98G cells using an ELISA-based STAT3 activation kit (Abcam). The results of these experiments confirmed that tacalcitol has a marked effect on STAT3 and dose-dependently suppresses STAT3 activation in T98G cells (Fig. 2F). In addition, we found that when we increased the concentration of 1α,25-dihydroxyvitamin D_3_ five-fold, there was a suppressive effect also by this compound. The results indicate that the effects on STAT3 activation is stronger by tacalcitol than by 1α,25-dihydroxyvitamin D_3_ (Fig. 2F). STAT3 is a driver of oncogenic transformation and the action of this protein is reported to be abnormal in a number of different types of cancers [[19], [20], [21]]. Thus, STAT3 is considered as one of the proteins of particular interest in the search for improved treatment of tumors, in the brain and elsewhere.

### 3.3 Tacalcitol and 1α,25-dihydroxyvitamin D_3_ suppress anchorage-independent growth of T98G cells

**Fig. 3:**
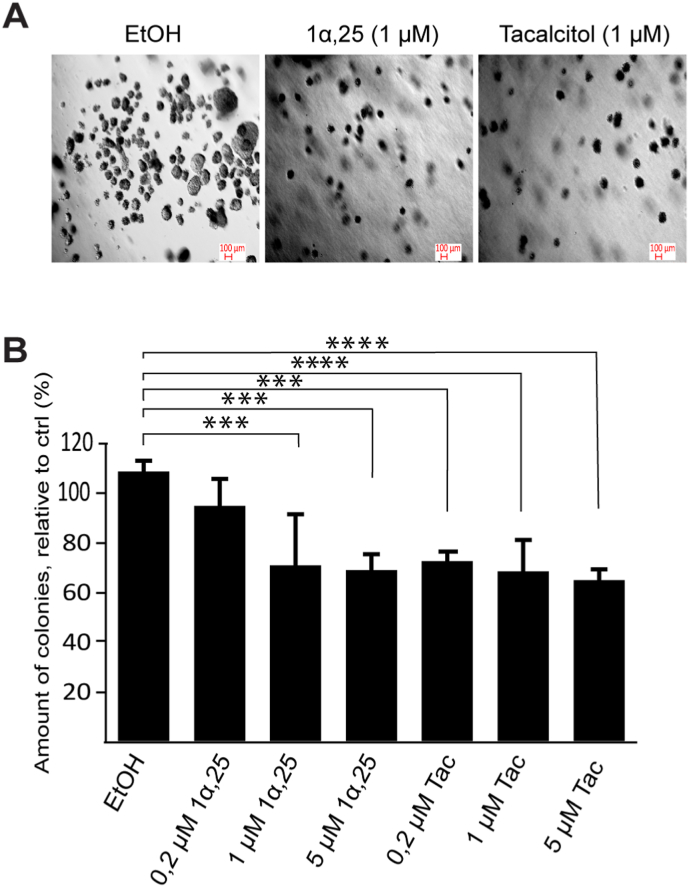
Effects of 1α,25-dihydroxyvitamin D_3_ and tacalcitol on anchorage-independent growth of T98G cells. (**A**) Representative images of colonies in wells treated with vehicle (EtOH) or 1 μM of 1α,25-dihydroxyvitamin D_3_ or tacalcitol. (**B**) Quantitative analysis of colonies formed on soft agar in the presence of vehicle or 0.2–5 μM of 1α,25-dihydroxyvitamin D_3_ (1α,25) or tacalcitol (Tac). The number of formed colonies with a diameter ≥70 μm were counted. The values represent mean ± standard deviation of 8–9 data points from three independent experiments, showing percentage of colonies relative to vehicle-treated cells. * = p < 0.05, ** = p < 0.01, *** = p < 0.001, **** = p < 0.0001.

As described, previous and present data in T98G cells show suppressive effects by tacalcitol and 1α,25-dihydroxyvitamin D_3_ in assays measuring growth, migration and oncogenic signaling. We wanted to examine if these compounds could alter the property of glioblastoma cells by which they can survive and grow without anchorage to the extracellular matrix. The ability of cancer cells for anchorage-independent growth enables them to expand and invade other tissues. Thus, we carried out soft agar colony formation assay, an established method for analysis of cellular transformation and anchorage-independent growth *in vitro* [22]. We found that both tacalcitol and 1α,25-dihydroxyvitamin D_3_ substantially suppressed soft agar colony formation in T98G cells (Fig. 3), indicating that these compounds may also be able to counteract the metastatic potential of glioblastoma cells.

### 3.4 Tacalcitol and 1α,25-dihydroxyvitamin D_3_ suppress STAT3 activation and anchorage-independent growth of U251 cells

**Fig. 4:**
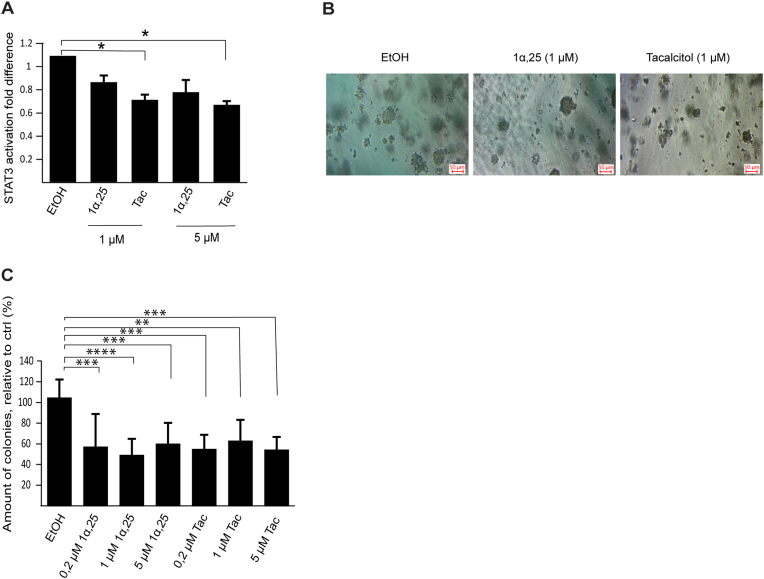
(**A**) Determination of STAT3 activation in U251 cells using a STAT3 assay kit. STAT3 activation was measured in cells cultured for 24 h in the absence (EtOH) or presence of 1α,25-dihydroxyvitamin D_3_ (1α,25) or tacalcitol (Tac) in the indicated concentrations. The values are presented as mean ± standard deviation of three independent determinations. (**B**) Effects of 1α,25-dihydroxyvitamin D_3_ and tacalcitol on anchorage-independent growth of U251 cells; representative images of colonies in wells treated with vehicle (EtOH) or 1 μM of 1α,25-dihydroxyvitamin D_3_ or tacalcitol. (**C**) Quantitative analysis of colonies in the presence of vehicle or 0.2–5 μM of 1α,25-dihydroxyvitamin D_3_ (1α,25) or tacalcitol (Tac) in U251 cells. The number of formed colonies with a diameter ≥40 μm were counted. The values represent mean ± standard deviation of 7–9 data points from three independent experiments. * = p < 0.05, ** = p < 0.01, *** = p < 0.001, **** = p < 0.0001.

To examine if the responses to 1α,25-dihydroxyvitamin D_3_ and tacalcitol are unique for T98G cells, we examined effects on STAT3 activation and anchorage-independent growth also in another VDR-expressing glioblastoma cell line, U251. Significant suppression of STAT3 signaling and anchorage-independent growth by these compounds was observed also in U251 cells (Fig. 4).

### 3.5 Effects of tacalcitol and 1α,25-dihydroxyvitamin D_3_ in glioblastoma cells; concluding remarks

As shown in our experiments, several properties of importance for growth and invasion of T98G glioblastoma cells are affected by 1α,25-dihydroxyvitamin D_3_ and its related analog tacalcitol. Although the effects by vitamin D and related compounds on growth and viability have been studied in a number of tumor cell types, there are few studies on the mechanisms for signaling by vitamin D analogs in glioblastoma. Ferronato et al. [23] studied effects of EM1, a vitamin D analog carrying an alkynylphosphonate moiety. For this compound, decreased levels of phosphorylated Akt and ERK were observed in glioblastoma cell lines including T98G [23]. Also, studies on proliferation have indicated effects by some vitamin D analogs, similarly as by vitamin D, on proteins associated with cell cycle arrest [23,24].

The development of vitamin D analogs for treatment of cancer is aimed to obtain compounds with tumor-suppressive effects resembling those of vitamin D while reducing toxicity associated with the high doses of vitamin D needed to efficiently suppress proliferation. Although vitamin D analogs are used clinically for the treatment of psoriasis and osteoporosis, there are currently no vitamin D analogs used clinically in cancer treatment despite promising findings in pre-clinical studies [13,14,25]. In order to construct analogs that are suitable and efficient enough for utilization as therapeutic agents in cancer, it is important to clarify how these synthetic molecules act in the cells. Also, it is important to explore structure-effect relationships of relevance for the cellular actions obtained. The present and previous data highlight the fact that the cellular mechanisms for effects by different vitamin D analogs can be quite unalike even though most, if not all, of these compounds are able to activate the VDR.

The present data indicate that several signaling proteins can be targets of vitamin D and vitamin D analogs in T98G cells. The strongest effects by tacalcitol were found on signaling proteins p70-S6 kinase and STAT3. Interestingly, signaling involving p70-S6 kinase is reported to play a key role in transformation of glial cells and inhibition of this protein has been shown to decrease tumor growth *in vivo* [26]. To the best of our knowledge, effects of vitamin D compounds on the p70-S6 kinase have not been previously shown in the brain and there is little information on vitamin D-related effects also for other tissues [27].

STAT3 has received a lot of interest as a potential target for cancer therapy due to its central role in regulation of various processes of importance in malignancy [19,21]. Previous studies in renal, colorectal and squamous carcinomas have shown suppression by 1α,25-dihydroxyvitamin D_3_ on STAT3 activation, indicating that STAT3 is a target for active vitamin D in several tissues [28,29]. In the current study we observed effects by both tacalcitol and 1α,25-dihydroxyvitamin D_3_ on STAT3 activation in T98G and U251 glioblastoma cells. However, the effect of tacalcitol was stronger, particularly in T98G cells. Thus, the small difference in structure between these two compounds (Fig. 1) is apparently enough to influence the effect. It should be noted that effects on PLCγ in T98G cells also appeared stronger for tacalcitol than for 1α,25-dihydroxyvitamin D_3_, whereas the effect on p70-S6 was about the same for both compounds.

Anchorage-independent growth is considered to correlate strongly with tumorigenicity and is an important property for invasion and metastasis in malignancy [30]. Our experiments on anchorage-independent growth in the glioblastoma cells showed substantial suppression by 1α,25-dihydroxyvitamin D_3_ as well as by tacalcitol. The potential links between the observed effects on signaling proteins and the inhibition of anchorage-independent growth are currently not clear. It may be surmised that inhibition of anchorage-independent growth, which is a complex phenomenon, may be dependent on more than one pathway where some of these pathways might be differentially regulated by different vitamin D compounds.

Potential contributing effects mediated via receptors other than the VDR (such as e. g. RORα/γ or AhR) and/or by hydroxyderivatives other than 1α,25-dihydroxyvitamin D_3_ also may play a role for vitamin D-related responses [[4], [5], [6], [31], [32]]. Our study shows similar effects in two glioblastoma cell lines, T98G and U251. However, vitamin D-related molecules may act on different cell types of the same cancer in different ways.

To summarize, the current results contribute to our understanding of effects by structurally similar vitamin D-related compounds in glioblastoma and underlines the potential for vitamin D analogs in future cancer therapy.

## Declaration of competing interest

The authors declare that they have no known competing financial interests or personal relationships that could have appeared to influence the work reported in this paper.
