# Supplementary material for: Effects of 1α,25-dihydroxyvitamin D3 and tacalcitol on cell signaling and anchorage-independent growth in T98G and U251 glioblastoma cells
Source: Biochem Biophys Rep. 2022 Jul 31;31:101313. doi: 10.1016/j.bbrep.2022.101313 (PMC9352528; doi:10.1016/j.bbrep.2022.101313)
Supplement: Multimedia component 1 [file mmc1.pdf]

Figure 1 pS473-Akt 60 kDa and AKT 60 kDa

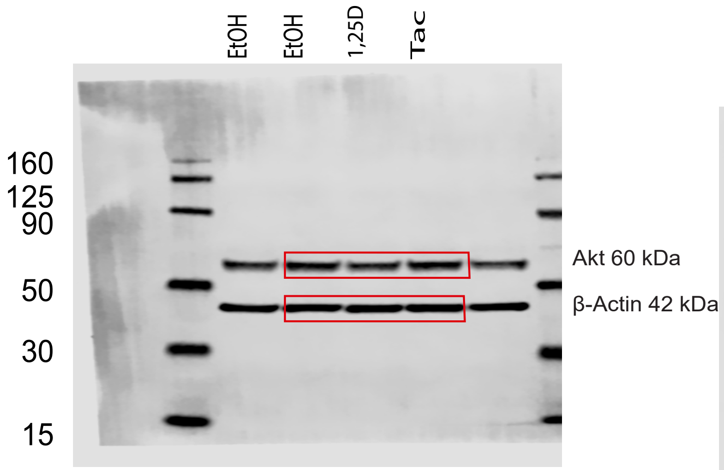

Repeat 1 channel 700 nm

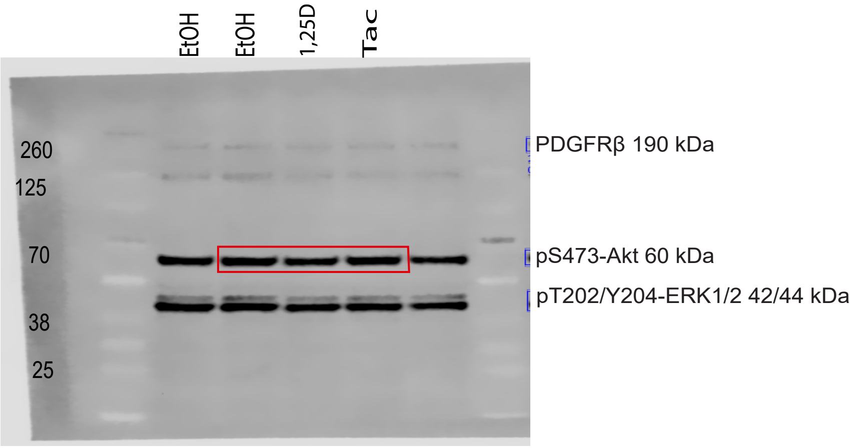

Repeat 1 channel 800 nm

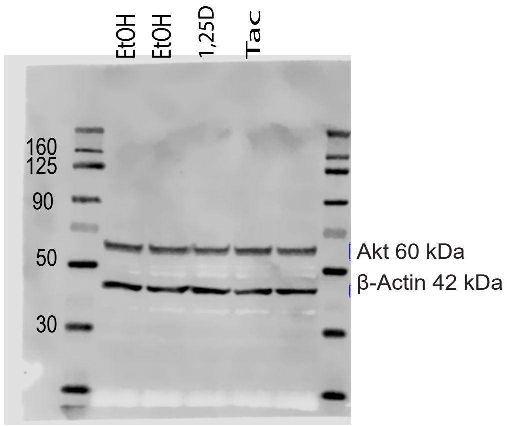

Repeat 2 channel 700 nm

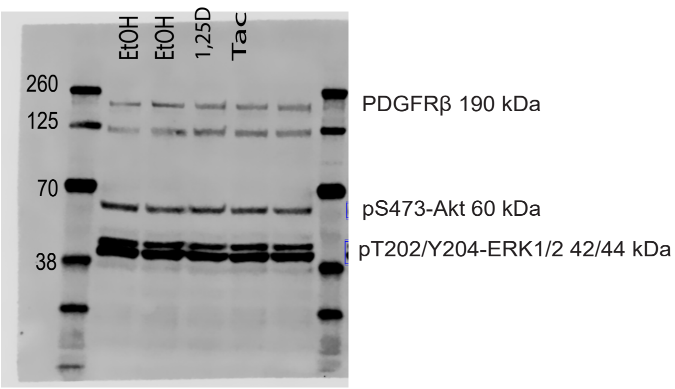

Repeat 2 channel 800 nm

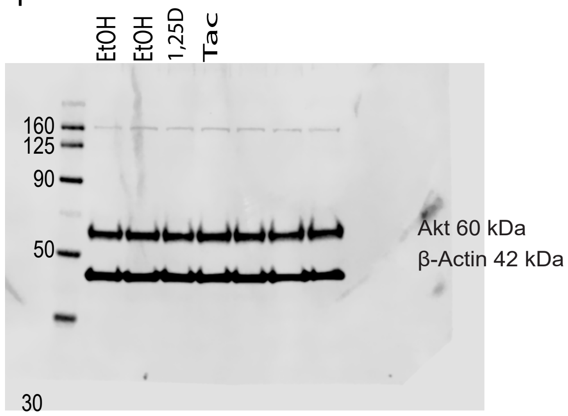

Repeat 3 channel 700 nm

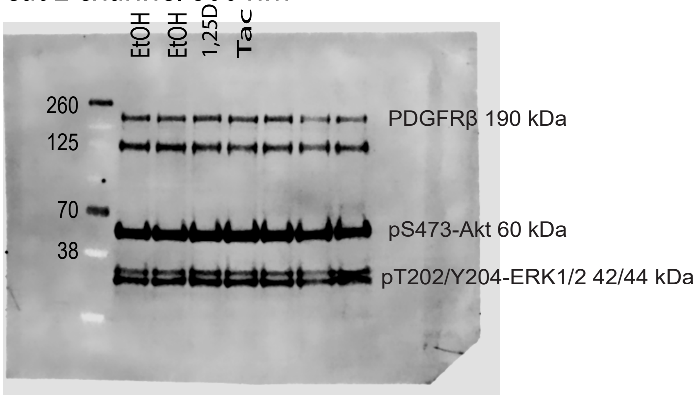

Repeat 3 channel 800 nm

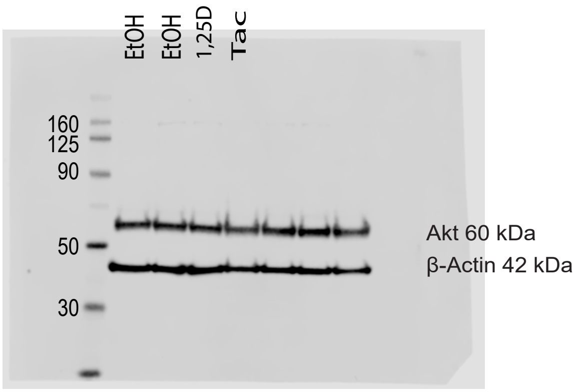

Repeat 4 channel 700 nm

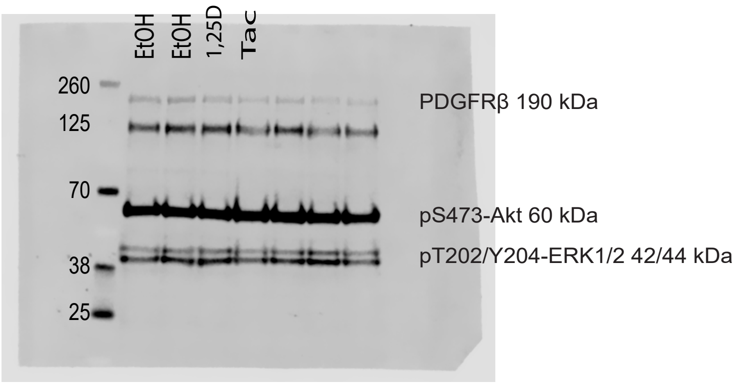

Repeat 4 channel 800 nm

Figure 1 uncropped blots pT202/Y204-ERK1/2

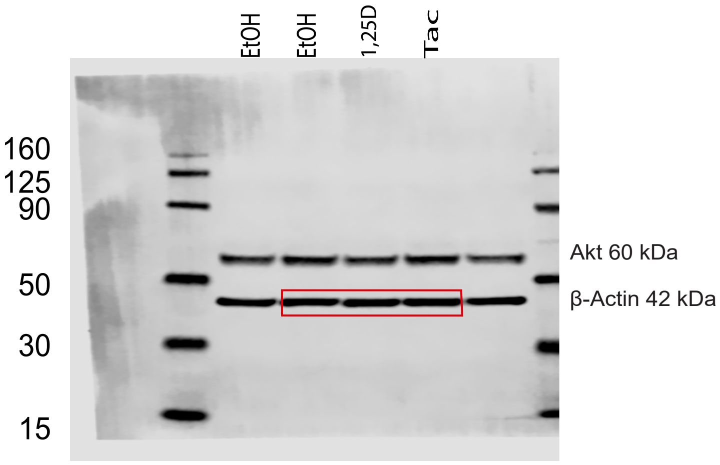

Repeat 1 channel 700 nm

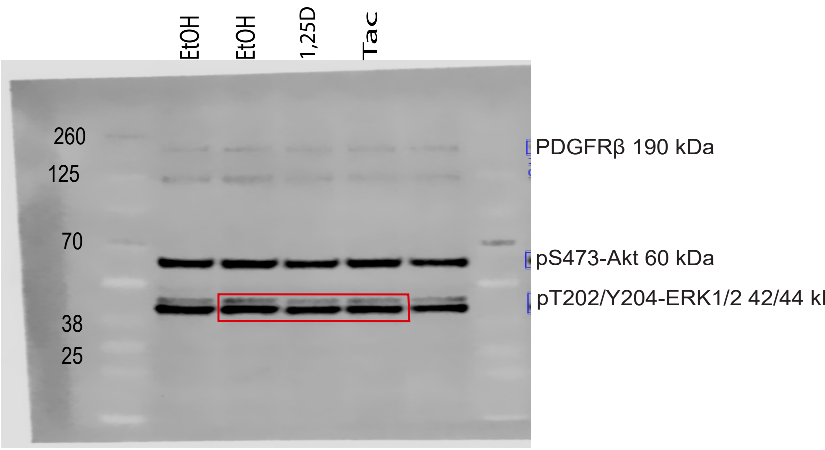

Repeat 1 channel 800 nm

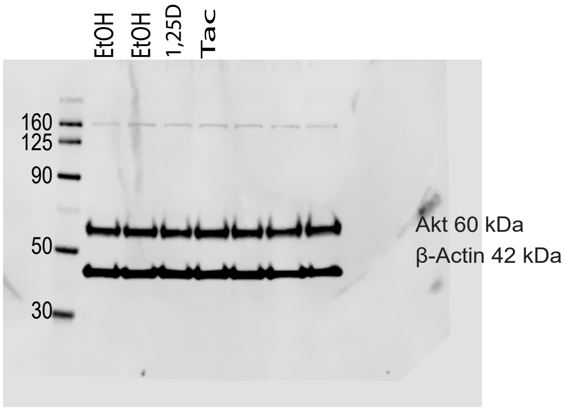

Repeat 2 channel 700 nm

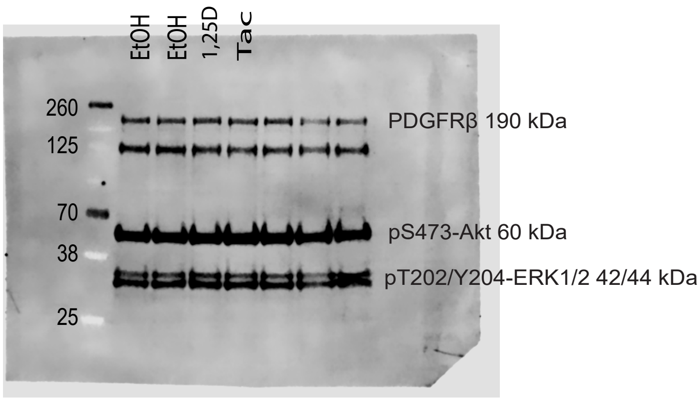

Repeat 2 channel 800 nm

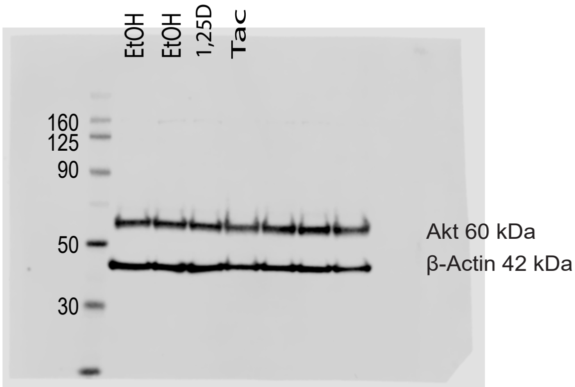

Repeat 3 channel 700 nm

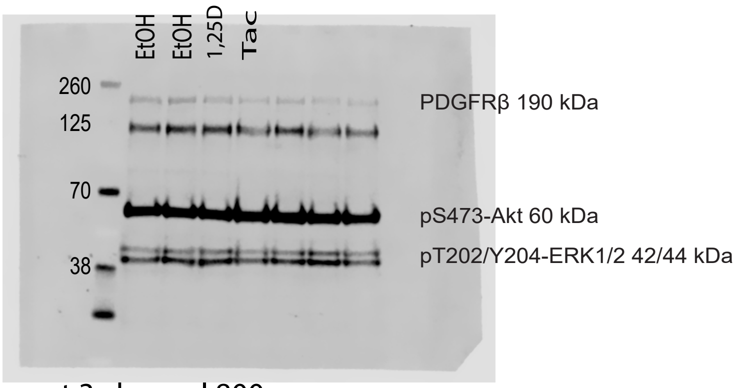

Repeat 3 channel 800 nm

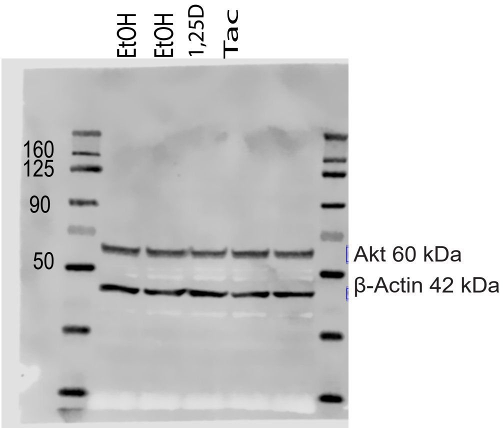

Repeat 4 channel 700 nm

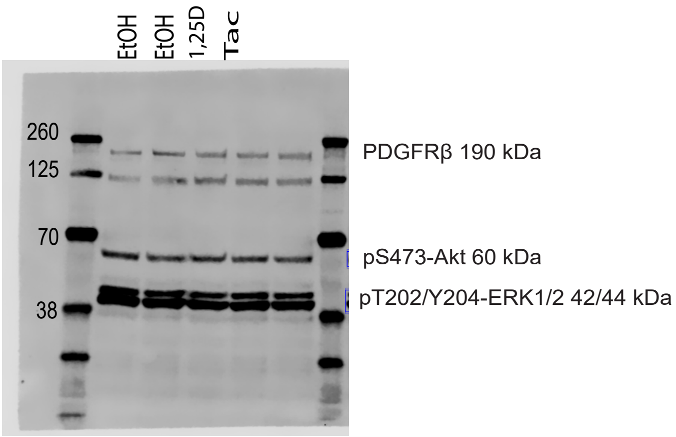

Repeat 4 channel 800 nm

Uncropped ERK1/2

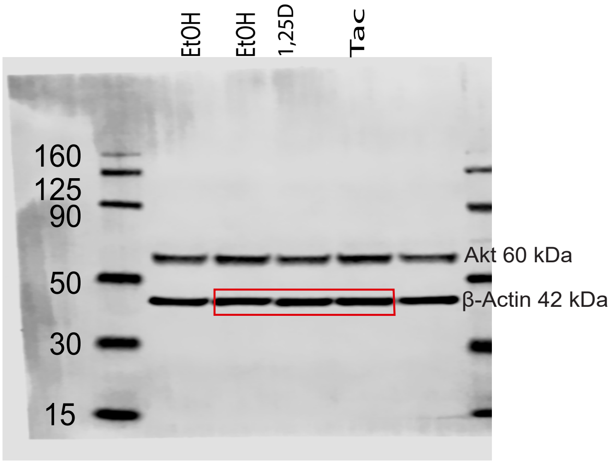

Repeat 1 channel 700 nm

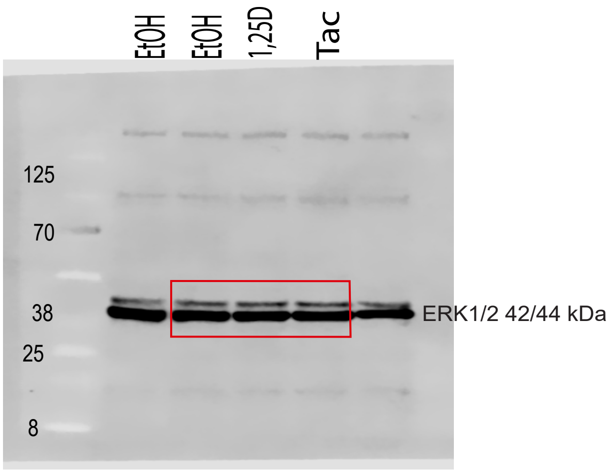

Repeat 1 channel 800 nm

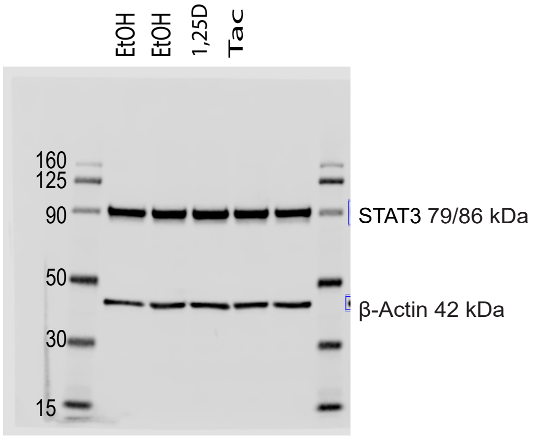

Repeat 2 channel 700 nm

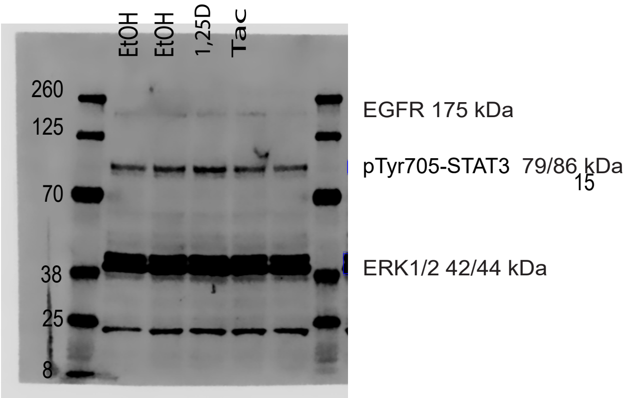

Repeat 2 channel 800 nm

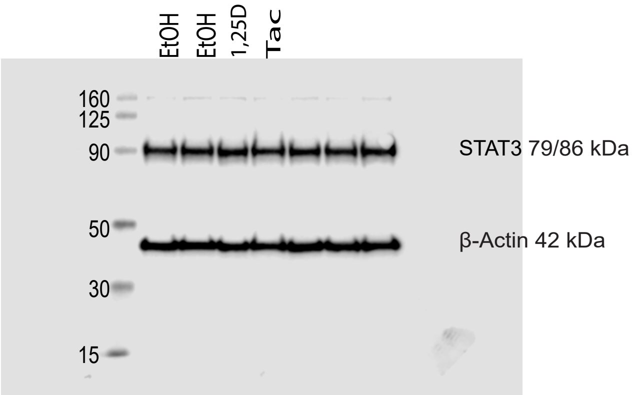

Repeat 3 channel 700 nm

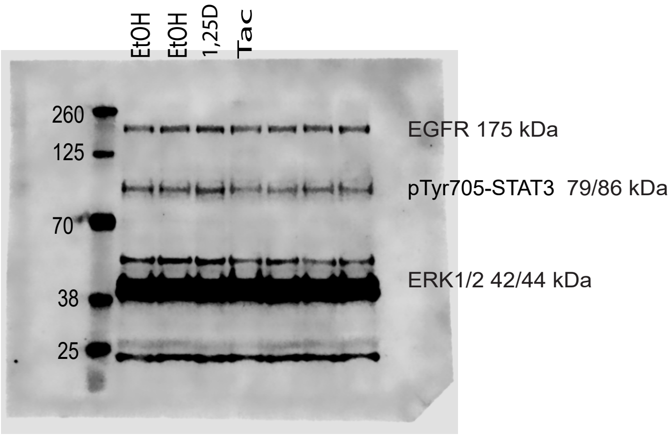

Repeat 3 channel 800 nm

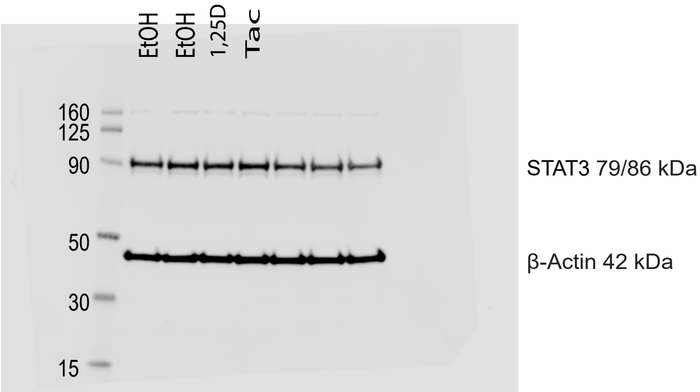

Repeat 4 channel 700 nm

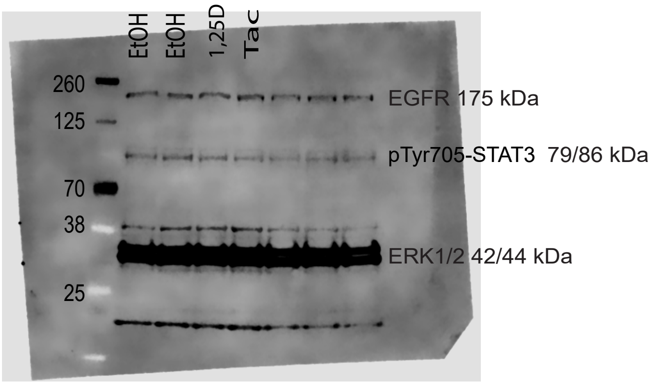

Repeat 4 channel 800 nm

Figure 1 p38 40 kDa

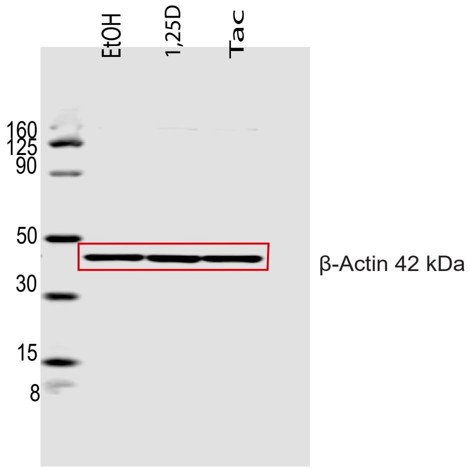

Repeat 1 channel 700 nm

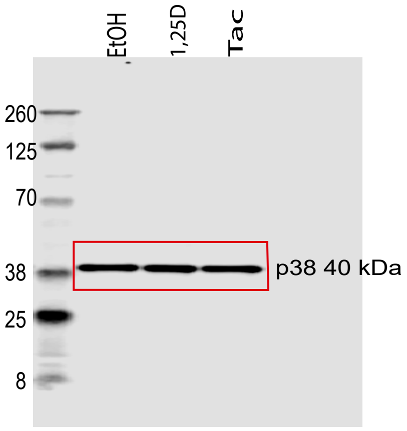

Repeat 1 channel 800 nm

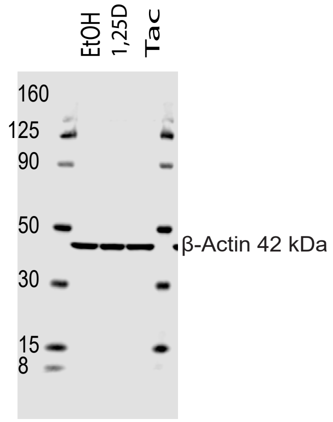

Repeat 2 channel 700 nm

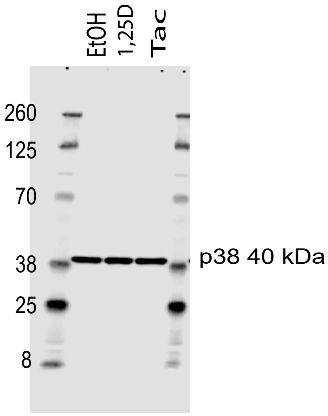

Repeat 2 channel 800 nm

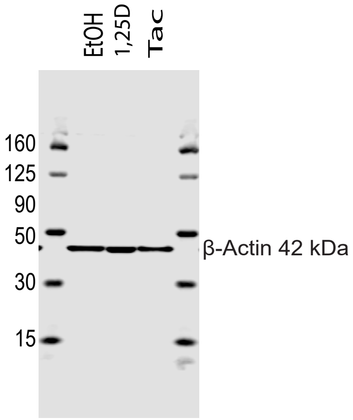

Repeat 3 channel 700 nm

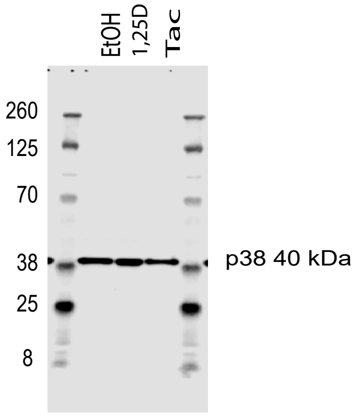

Repeat 3 channel 800 nm
