# Supplementary material for: Effects of 1α,25-dihydroxyvitamin D3 and tacalcitol on cell signaling and anchorage-independent growth in T98G and U251 glioblastoma cells
Source: Biochem Biophys Rep. 2022 Jul 31;31:101313. doi: 10.1016/j.bbrep.2022.101313 (PMC9352528; doi:10.1016/j.bbrep.2022.101313)
Supplement: Multimedia component 2 [file mmc2.pdf]

Figure 2 pY783- PLCγ 155 kDa and PLCγ 155 kDa

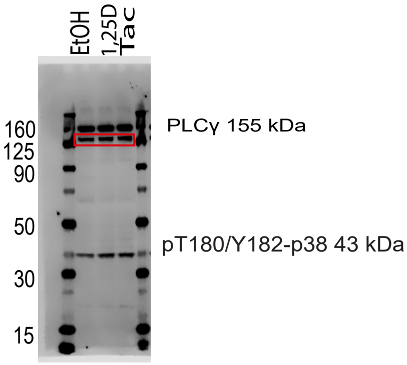

Repeat 1 channel 700 nm

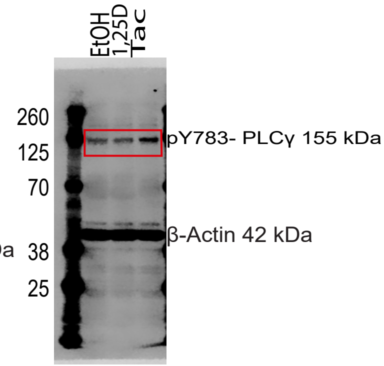

Repeat 1 channel 800 nm

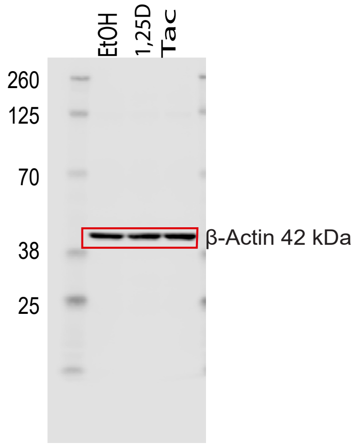

Repeat 1 channel 800 nm

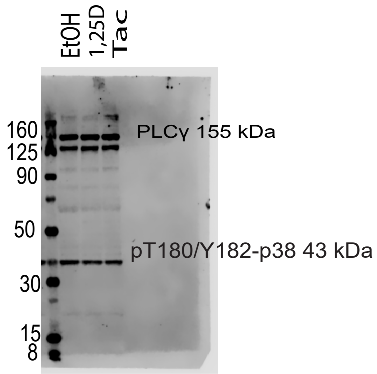

Repeat 2 channel 700 nm

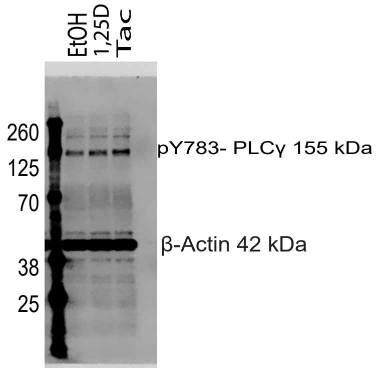

Repeat 2 channel 800 nm

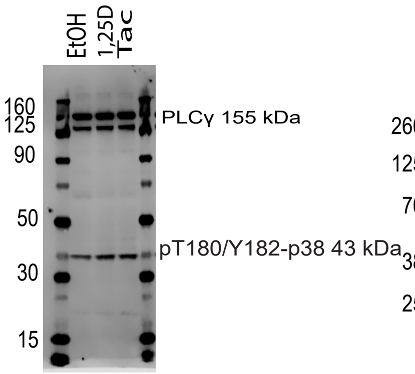

Repeat 3 channel 700 nm

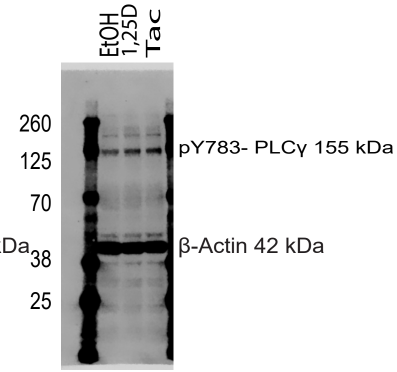

Repeat 3 channel 800 nm

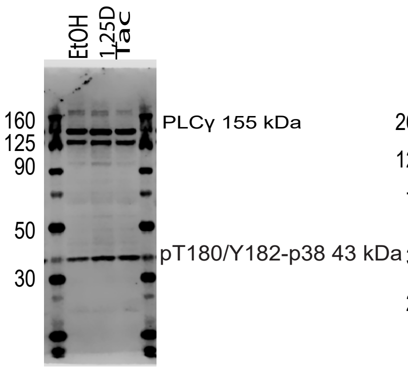

Repeat 4 channel 700 nm

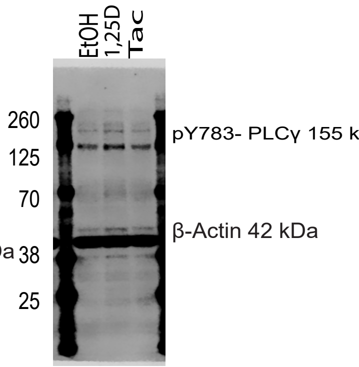

Repeat 4 channel 800 nm

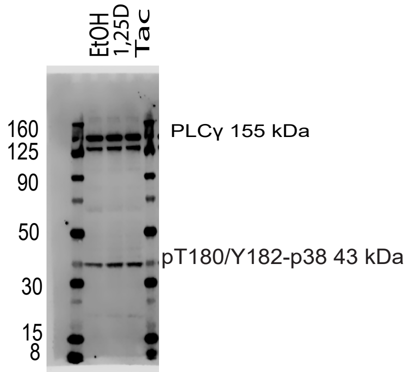

Repeat 5 channel 700 nm

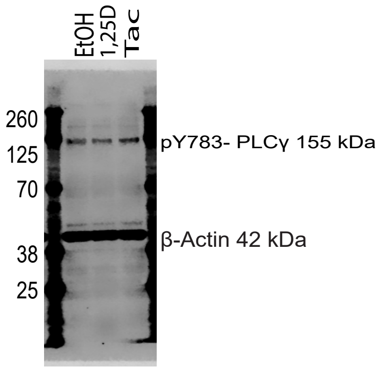

Repeat 5 channel 800 nm

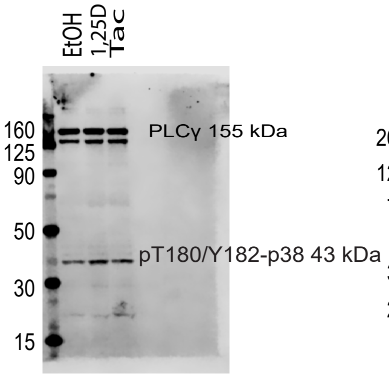

Repeat 6 channel 700 nm

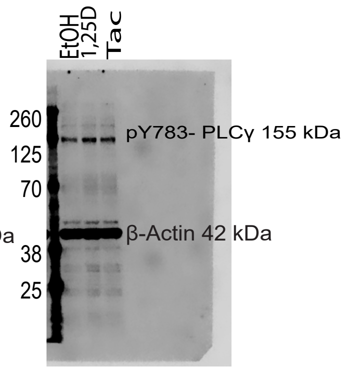

Repeat 6 channel 800 nm

Figure 2 pT389-p70 S6 Kinase 70 kDa

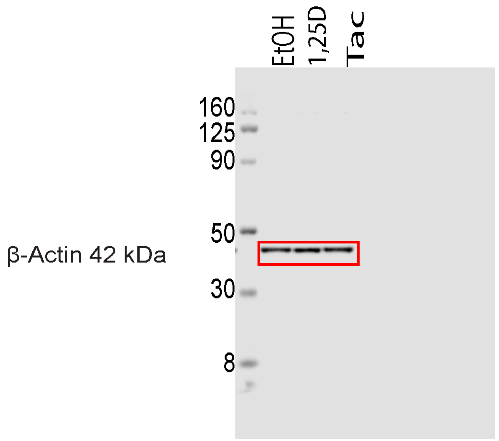

Repeat 1 channel 700 nm membrane 1

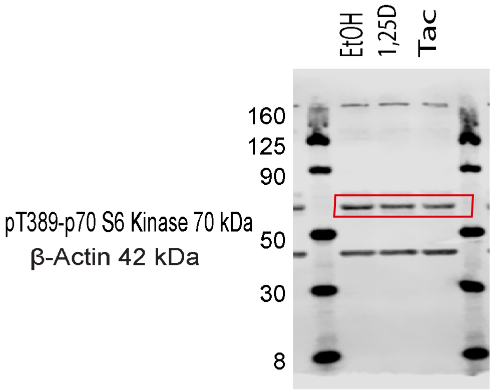

Repeat 1 channel 700 nm membrane 2

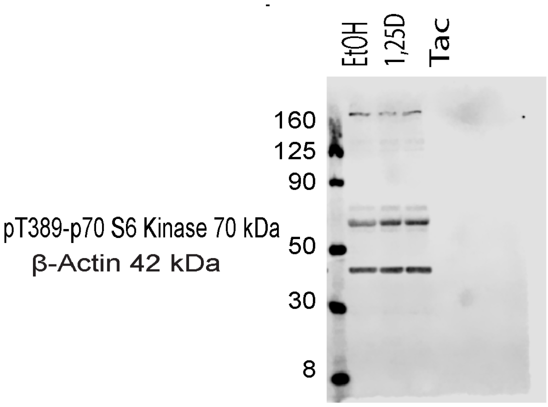

Repeat 2 channel 700 nm

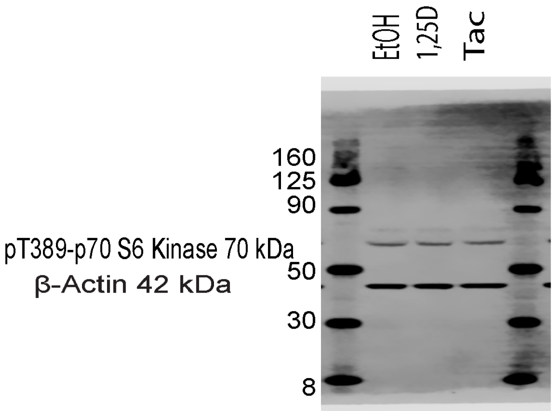

Repeat 3 channel 700 nm

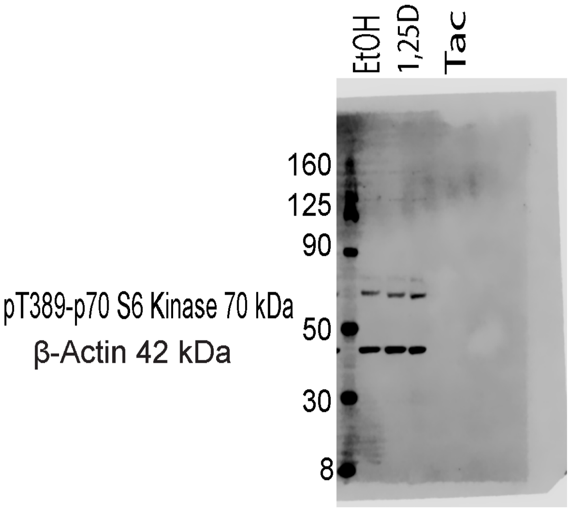

Repeat 4 channel 700 nm

Figure 2 p70 S6 Kinase 70 kDa

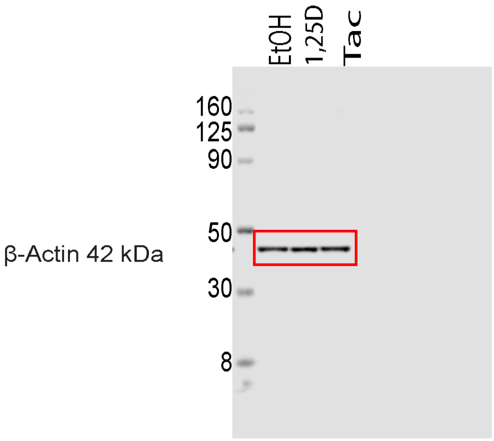

Repeat 1 channel 700 nm

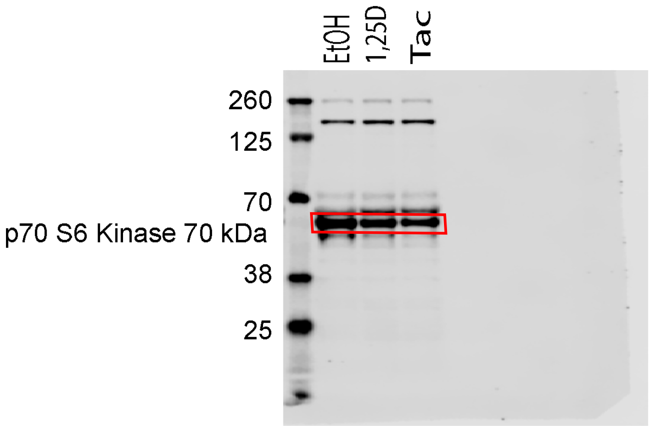

Repeat 1 channel 800 nm

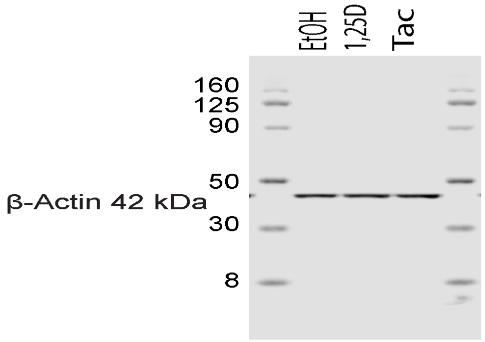

Repeat 2 channel 700 nm

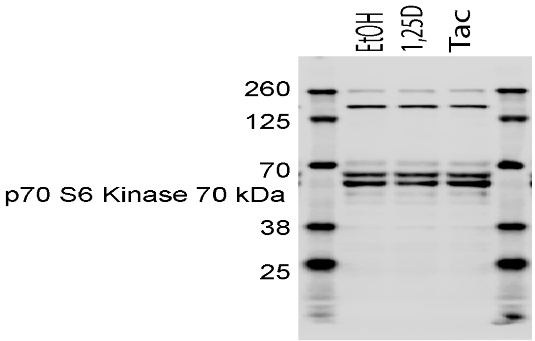

Repeat 2 channel 800 nm

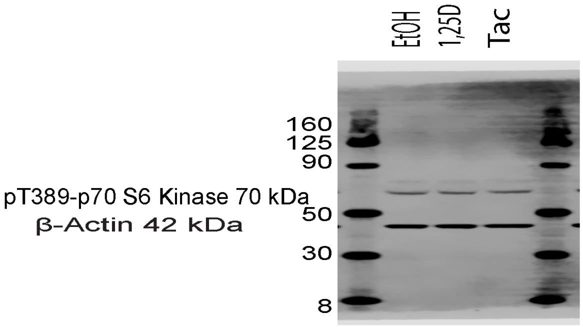

Repeat 3 channel 700 nm

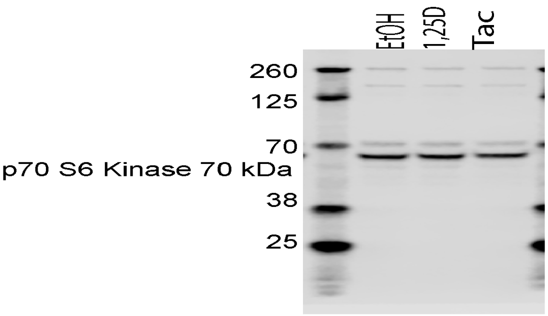

Repeat 3 channel 800 nm

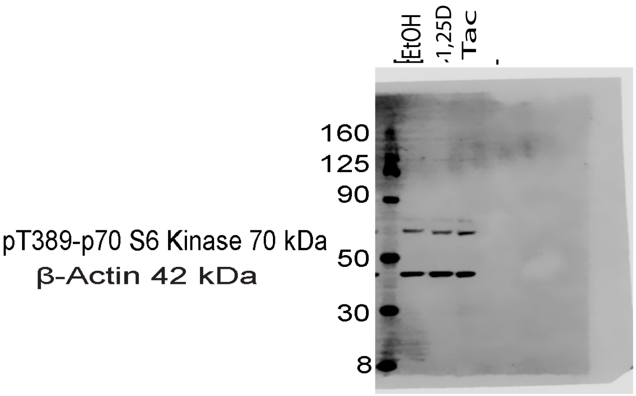

Repeat 4 channel 700 nm

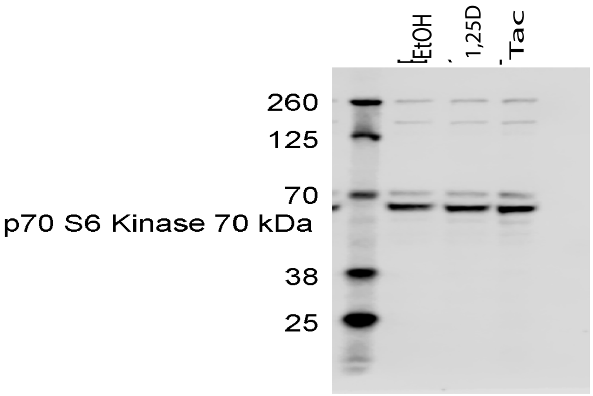

Repeat 4 channel 800 nm

Figure 2 pTyr705-STAT3 79/86 kDa and STAT3 79/86 kDa

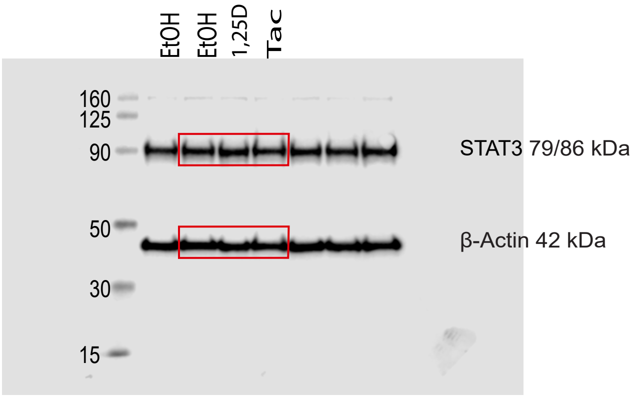

Repeat 1 channel 700 nm

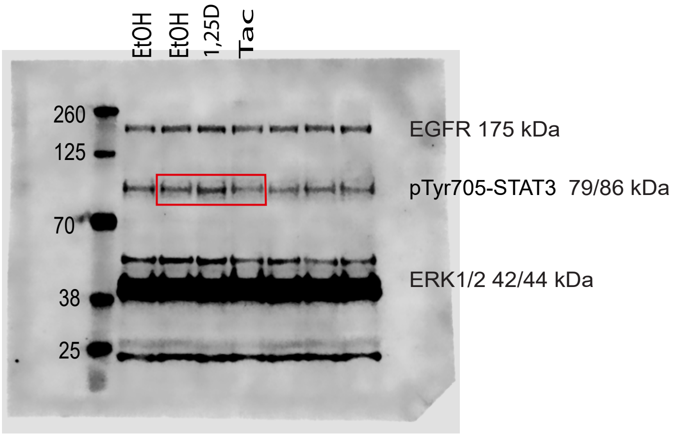

Repeat 1 channel 800 nm

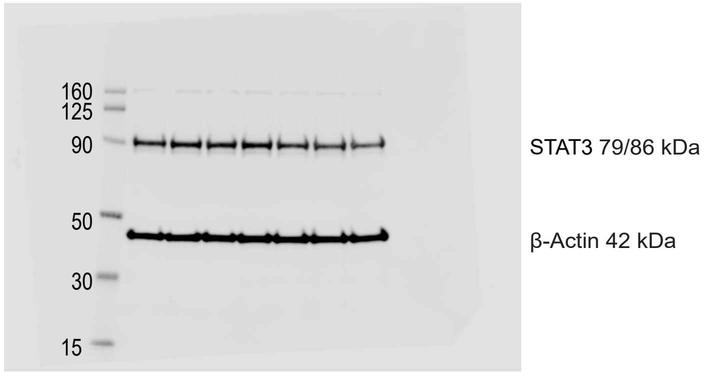

Repeat 2 channel 700 nm

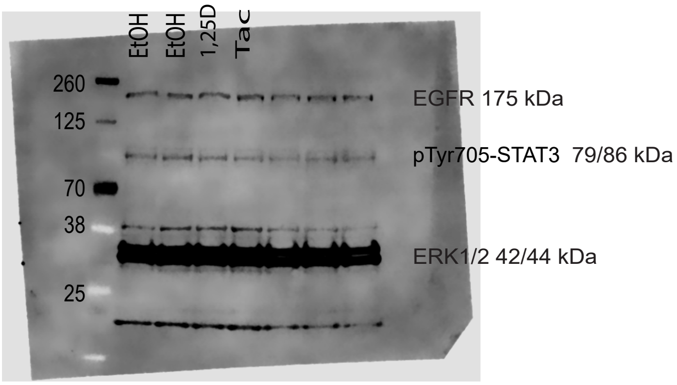

Repeat 2 channel 800 nm

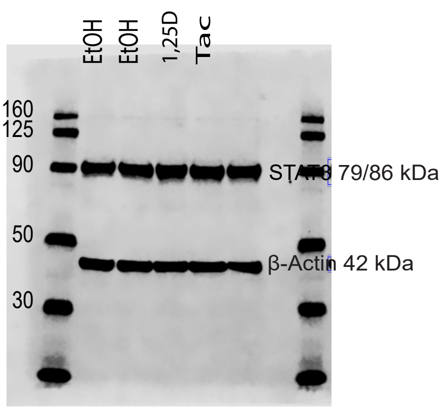

Repeat 3 channel 700 nm

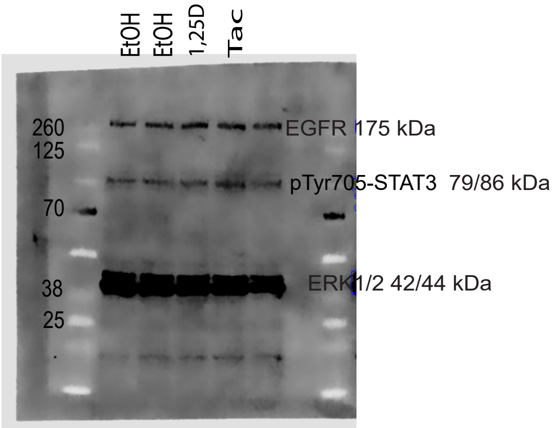

Repeat 3 channel 800 nm

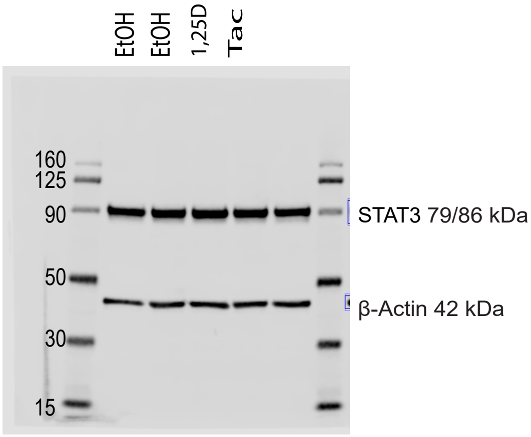

Repeat 4 channel 700 nm

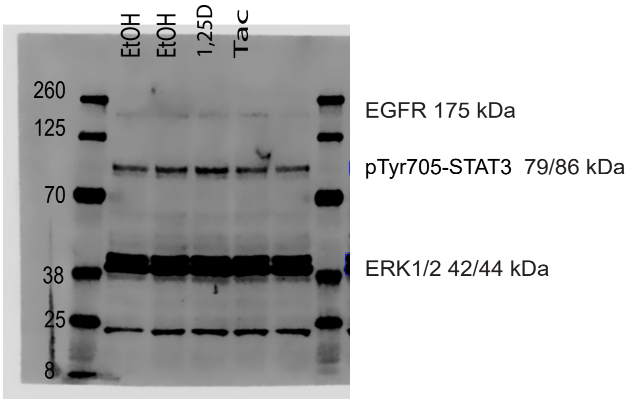

Repeat 4 channel 800 nm
